# Lung necrosis and neutrophils reflect common pathways of susceptibility to *Mycobacterium tuberculosis* in genetically diverse, immune-competent mice

**DOI:** 10.1242/dmm.020867

**Published:** 2015-09-01

**Authors:** Muhammad K. K. Niazi, Nimit Dhulekar, Diane Schmidt, Samuel Major, Rachel Cooper, Claudia Abeijon, Daniel M. Gatti, Igor Kramnik, Bulent Yener, Metin Gurcan, Gillian Beamer

**Affiliations:** 1 Department of Biomedical Informatics, The Ohio State University, Columbus, 43210 OH, USA; 2 Department of Computer Science and Department of Electrical, Computer and Systems Engineering, Rensselaer Polytechnic Institute, Troy, 12810 NY, USA; 3 Department of Infectious Disease and Global Health, Cummings School of Veterinary Medicine, Tufts University, Grafton, 01536 MA, USA; 4 The Jackson Laboratory, Bar Harbor, 04662 ME, USA; 5 Department of Medicine, Boston University School of Medicine, Boston, 02215 MA, USA

**Keywords:** Tuberculosis, *Mycobacterium tuberculosis*, Neutrophils, Necrosis, CXCL1, CXCL2, CXCL5, Machine learning, Diversity outbred, DO

## Abstract

Pulmonary tuberculosis (TB) is caused by *Mycobacterium tuberculosis* in susceptible humans*.* Here, we infected Diversity Outbred (DO) mice with ∼100 bacilli by aerosol to model responses in a highly heterogeneous population. Following infection, ‘supersusceptible’, ‘susceptible’ and ‘resistant’ phenotypes emerged. TB disease (reduced survival, weight loss, high bacterial load) correlated strongly with neutrophils, neutrophil chemokines, tumor necrosis factor (TNF) and cell death. By contrast, immune cytokines were weak correlates of disease. We next applied statistical and machine learning approaches to our dataset of cytokines and chemokines from lungs and blood. Six molecules from the lung: TNF, CXCL1, CXCL2, CXCL5, interferon-γ (IFN-γ), interleukin 12 (IL-12); and two molecules from blood – IL-2 and TNF – were identified as being important by applying both statistical and machine learning methods. Using molecular features to generate tree classifiers, CXCL1, CXCL2 and CXCL5 distinguished four classes (supersusceptible, susceptible, resistant and non-infected) from each other with approximately 77% accuracy using completely independent experimental data. By contrast, models based on other molecules were less accurate. Low to no IFN-γ, IL-12, IL-2 and IL-10 successfully discriminated non-infected mice from infected mice but failed to discriminate disease status amongst supersusceptible, susceptible and resistant *M.-tuberculosis-*infected DO mice. Additional analyses identified CXCL1 as a promising peripheral biomarker of disease and of CXCL1 production in the lungs. From these results, we conclude that: (1) DO mice respond variably to *M. tuberculosis* infection and will be useful to identify pathways involving necrosis and neutrophils; (2) data from DO mice is suited for machine learning methods to build, validate and test models with independent data based solely on molecular biomarkers; (3) low levels of immunological cytokines best indicate a lack of exposure to *M. tuberculosis* but cannot distinguish infection from disease.

## Abstract (precis)

**Summary:** Molecular biomarkers of tuberculosis are identified and used to classify disease status of Diversity Outbred mice that have been infected with *Mycobacterium tuberculosis*.

## INTRODUCTION

Over the past two decades, inbred laboratory mice have been used to identify important host responses to *Mycobacterium tuberculosis*. However, studies using inbred mice might not generalize to all mice or to all humans. Benefits of using a diverse experimental population are the reduction of erroneous interpretations due to strain-specific effects and the detection of new phenotypes that are not present in standard inbred laboratory strains (Yang et al., 2007, 2011). Therefore, we have started using Diversity Outbred (DO) mice as another tool to understand susceptibility to *M. tuberculosis*. The genetic diversity of the DO population is on par with that of the human population, and the DO genomes are suited for high-resolution genetic mapping. For these reasons, DO mice have been used to identify novel genetic associations with a variety of traits (Harrison et al., 2009; Churchill et al., 2012; Svenson et al., 2012; Ferris et al., 2013; Logan et al., 2013; Recla et al., 2014), but they have not yet been used extensively as a model of *M. tuberculosis* infection.

Heterogeneity in the DO population is due to eight distinctive founder strains that were chosen for maximal genetic diversity. Five founder strains are inbred laboratory strains and three are wild-derived strains (Churchill et al., 2012; Svenson et al., 2012). Only three of the eight founder strains (C57BL/6, 129 and A/J) have been used in *M. tuberculosis* research, primarily used to identify requirements for TH1-mediated immunological resistance and to provide some insight into susceptibility (Medina and North, 1998; Jagannath et al., 2000; Chackerian and Behar, 2003; Beamer and Turner, 2005). *M. tuberculosis* infection of the other five founder strains (NOD/LtJ, NZO/HlLtJ, CAST/EiJ, PWK/PhJ, and WSB/EiJ) has not yet been reported. To our knowledge, DO mice have been used in only one *M. tuberculosis* infection study (Gopal et al., 2013) and one aging study (Harrison et al., 2014). Thus, additional work is needed. Here, we infected DO mice with *M. tuberculosis* by aerosol and evaluated morbidity, bacterial burden and a set of immunological and inflammatory molecules in lungs, antigen-stimulated blood cultures and plasma. Multiple methods (simple and complex statistical analyses, and machine learning) were used to identify and rank important molecular features. The features were then used to generate tree classification models to discriminate disease status using only molecular biomarkers. Finally, tree classification models were tested using data from a completely independent experiment.

Decades of research show a complex host response to *M. tuberculosis*, involving many cell types, molecules and signaling pathways. Resistance to mycobacterial infection in humans, and to *M. tuberculosis* in mice, requires TH1 polarized cell-mediated immunity, including the cytokines IFN-γ, IL-12, TNF and IL-2. Additionally, tuberculosis (TB) disease might be accelerated by the immune suppressive cytokine IL-10. When absent or blocked, these cytokines alter the survival of mice, indicating that their presence is important for outcome (Gong et al., 1996; Flynn and Chan, 2001; Turner et al., 2002; Beamer et al., 2008a,b; Cooper, 2009; Higgins et al., 2009; Zhang et al., 2011). Similarly, humans with TH1 deficiencies are at increased risk of mycobacterial disease (Casanova and Abel, 2002; Redford et al., 2011; Liang et al., 2014), and there is evidence that IL-10 is associated with pulmonary TB in humans as well (Gong et al., 1996; Zhang et al., 2011).

**TRANSLATIONAL IMPACT**

**Clinical issue**

Tuberculosis (TB) is an infectious disease caused by *Mycobacterium tuberculosis*, which typically affects the lungs. Most infections remain latent, and only about 10% of infected individuals develop active TB, which, if left untreated, can be fatal. Animal models are a useful research tool to understand mechanisms that determine susceptibility to *M. tuberculosis*.  This work models TB in a genetically heterogeneous experimental mouse population, called Diversity Outbred mice, which might better model the human population than standard inbred strains of mice that are commonly used in biomedical research.

**Results**

The results show that some immune responses and disease features in Diversity Outbred mice reflect disease features of human pulmonary TB – including lung and granuloma necrosis, and the presence of neutrophils. Furthermore, the authors developed and tested models that could discriminate disease status with 77% accuracy based on the detection of three neutrophil chemokines: CXCL1, CXCL2 and CXCL5. One of these chemokines, CXCL1, has the most potential to be a peripheral plasma biomarker of lung disease and of CXCL1 lung levels in individuals with TB. Additionally, as in humans, pro-inflammatory, cell-mediated, TH1 antigen-specific immune responses do not reflect TB disease state very well, but low immunological responses are very useful to identify non-exposed individuals.

**Implications and future directions**

This work is expected to influence the field, by showing that Diversity Outbred mice can model pathologic changes observed in human pulmonary TB, and therefore can be useful to identify and understand mechanisms of necrosis and neutrophil involvement in human TB. Future work using these mice will be exceptionally valuable when larger data sets are generated both for genetic studies, aimed at identifying novel genes and loci associated with increased susceptibility, and for longitudinal biomarker studies, in particular those focused on markers and models that can predict outcome before disease ensues.

A perplexing problem is that pulmonary TB, the contagious form of TB, most commonly occurs in immune-competent individuals who remain immunologically responsive to mycobacterial antigens and do not have TH1 defects (Hunter et al., 2006; Modlin and Bloom, 2013; Andersen and Woodworth, 2014; Nunes-Alves et al., 2014; Vilaplana and Cardona, 2014). Thus, although TH1 immunity is a requirement for resistance, its presence does not prevent disease in susceptible individuals. For many years, lung and granuloma necrosis, and neutrophils have been recognized as negative indicators of pulmonary TB in humans (Barnes et al., 1988; Berry et al., 2010; Leong et al., 2011). In fact, many studies implicate neutrophils or neutrophil-like cells, and chemokines that recruit neutrophils, as causal mediators of lung damage in mice (Keller et al., 2006; Lyadova et al., 2010; Nandi and Behar, 2011; Yang et al., 2012; Gopal et al., 2013; Major et al., 2013; Dorhoi et al., 2014; Nouailles et al., 2014). Therefore, we assessed necrosis, neutrophils and the neutrophil chemokines CXCL1, CXCL2 and CXCL5 to determine whether these markers of TB disease show phenotypic variation in *M.-tuberculosis-*infected DO mice.

Following aerosol infection, DO mice indeed varied in survival, morbidity, bacterial burden, granuloma composition, immunological cytokines and neutrophil chemokines. Nearly half of the DO mouse population rapidly developed morbidity associated with necrosis of lung tissue and granulomas, with neutrophil influx. Strong and significant disease correlates were lung neutrophil chemokines (CXCL1, CXCL2, CXCL5), TNF and dead cells. By contrast, IL-12, IL-2, IFN-γ and IL-10 were weak, or were not, disease correlates. In the periphery (plasma), only CXCL1 was a strong and statistically significant correlate of disease and of lung CXCL1, whereas CXCL5 was not, and neither were any other cytokines.

Statistical analyses, data mining and machine learning approaches identified important molecular features that were then used to classify status of *M.-tuberculosis-*infected mice in the absence of disease indicators (survival, weight loss, bacterial burden). Lung TNF, CXCL1, CXCL2, CXCL5, IFN-γ, IL-12 and blood IL-2 and TNF were consistently identified as important. The most accurate models relied on CXCL1, CXCL2 and CXCL5 to classify ‘supersusceptible’, ‘susceptible’, ‘resistant’ and ‘non-infected’ mice. TNF was an important feature, but models that included TNF were less accurate because they confused resistant mice with supersusceptible mice. Immune cytokines were highly successful in discriminating non-infected from infected mice, but could not distinguish disease status.

In summary, of the molecules that we measured, neutrophil chemokines CXCL1, CXCL2 and CXCL5 best distinguished TB disease in highly susceptible DO mice, whereas low and/or a lack of immune cytokines identified non-infected individuals. Furthermore, the classification model performed well using completely independent experimental data, resulting in 77% accuracy. Similar to humans, necrosis and neutrophils are TB disease features, and CXCL1 is a good peripheral biomarker in DO mice. (Gopal et al., 2013). Overall, these results indicate DO mice are another useful murine model of pulmonary TB, in particular to understand the mechanisms that contribute to neutrophil recruitment and necrosis during early *M. tuberculosis* infection.

## RESULTS

### DO mice respond variably to atomized *M. tuberculosis*

**Fig. 1.**
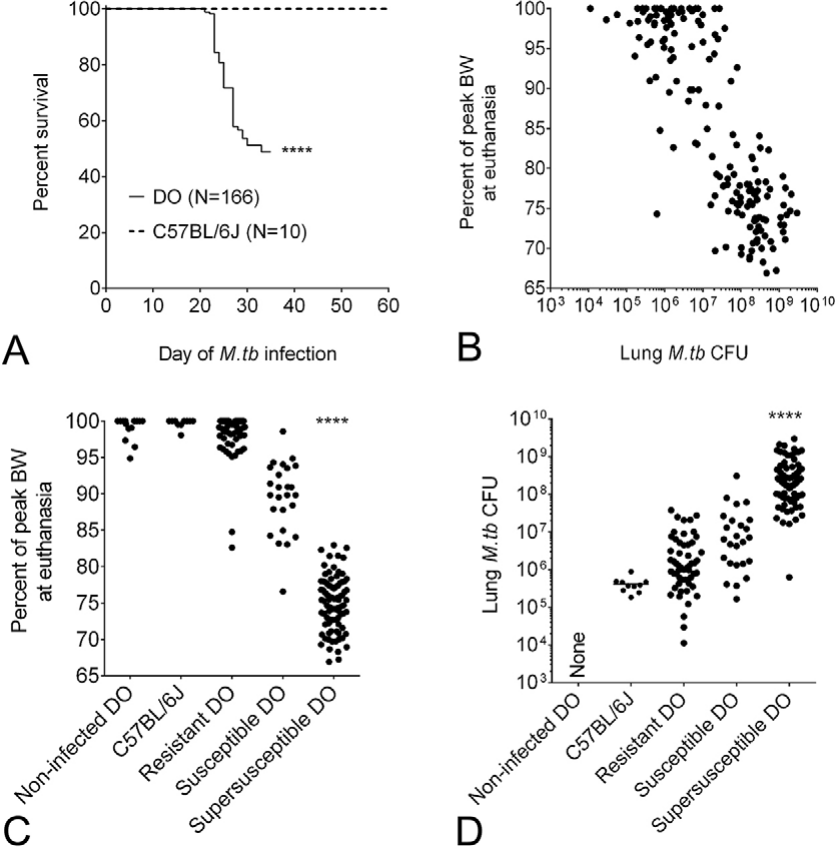
***M. tuberculosis* infection and TB disease indicators in mice.** Female 8-week-old non-sibling DO mice (*N*=166) and C57BL/6J mice (*N*=10) were infected with ∼100 *M. tuberculosis* (*M*.*tb*) bacilli by aerosol. Survival was assessed by euthanasia owing to morbidity or by euthanasia at day 35 of infection, whichever came first. Survival of DO mice compared with that of the parental C57BL/6J strain was analyzed by using a Log-rank test, ****P<0.001 (A). The percentage of peak body weight (BW) and lung *M. tuberculosis* CFU were strong, significant negative correlations in DO mice (Spearman r −0.79, *P*<0.001, 95% CI −0.84 to −0.72) (B). The percentage of peak BW (C) and lung *M. tuberculosis* burden (D) from non-infected, resistant, susceptible and supersusceptible (described in the Materials and Methods) DO mice, and C57BL/6J mice were analyzed by using ANOVA with Tukey's post-test, ****P<0.0001. Data are combined from two independent experimental infections: *N*=97 DO mice in the first experiment, *N*=69 DO mice and *N*=10 C57BL/6J mice in the second experiment.

All mice gained weight for at least two weeks following infection with ∼100 *M. tuberculosis* bacilli (supplementary material Fig. S1). Afterwards, nearly half of the DO mice developed morbidity requiring euthanasia before 35 days, resulting in significantly reduced survival compared to the founder C57BL/6J strain (Fig. 1A). Reduced survival strongly reflected the daily rate of weight loss (not shown), as observed in other heterogeneous mice (Harrison et al., 2014). As expected, the percentage of the peak body weight at euthanasia and lung *M. tuberculosis* burden had a strong, significant inverse correlation (Fig. 1B) such that high bacterial burden associated with weight loss. Interestingly, these effects were independent of absolute body weight before infection or at peak body weight (not shown). Thus, body weight alone does not appear to protect against TB.

**Fig. 2.**
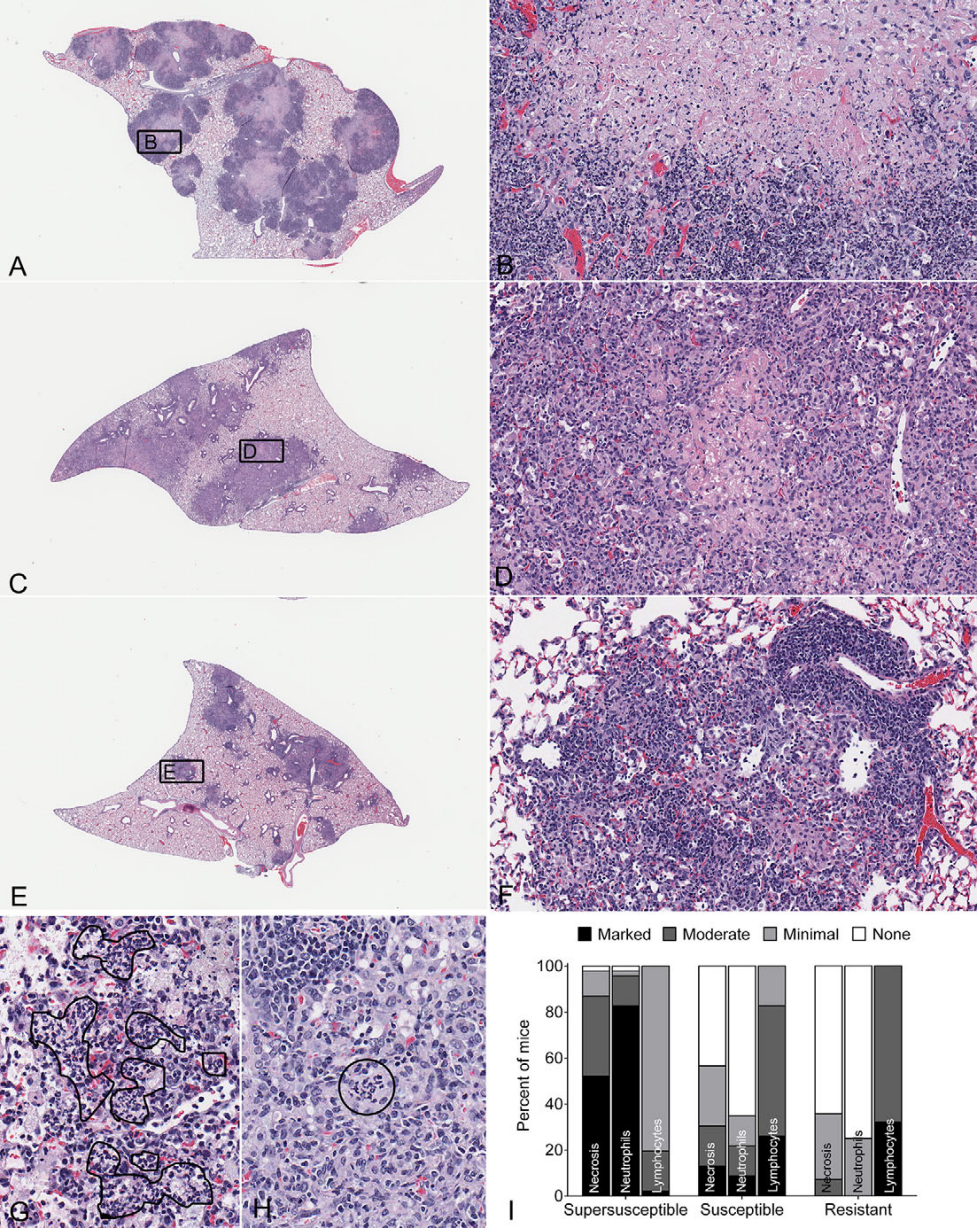
**Lung lesions in *M.-tuberculosis*-infected DO mice.** Female 8-week-old non-sibling DO mice (*N*=97) were infected with ∼100 *M. tuberculosis* (*M*.*tb*) bacilli by using an aerosol. Representative hematoxylin and eosin (H&E)-stained lung sections are shown. Supersusceptible (A) with inset showing substantial lung tissue, macrophage and neutrophil necrosis with capillary thrombosis magnified (B); susceptible (C) with inset showing a small region of necrosis surrounded by inflammatory cells that lacks thrombosis (D); resistant (E) with inset showing a small, non-necrotic lesion with an abundance of perivascular lymphocytes (F). Black lines surround neutrophils in alveolar spaces (G) and within macrophage-rich granulomas (H). Magnifications are 10× (A,C,E); 200× (B,D,F); and 400× (G,H). Two lung lobes from each mouse were scored for relative severity of each lesion type by a board-certified veterinary pathologist (G.B.) without knowledge of the groups, and the data was compiled (I).

It is common in human TB studies to stratify individuals by using clinical symptoms or disease severity, and we applied this approach to DO mice. Three susceptibility classes were detected during infection – supersusceptible, susceptible and resistant. Fig. 1C,D show that supersusceptible mice lost the most body weight and had the highest lung bacterial burdens; susceptible mice were intermediate for both; and resistant mice exhibited the lowest values for both. Fig. 2 shows common lung lesions for each susceptibility class. The lungs of supersusceptible DO mice typically contained large lesions, occupying approximately 75% of lung tissue, which were effaced by coalescing foci with central coagulation necrosis of lung alveolar septae and intra-alveolar macrophages [resembling caseous necrosis (Leong et al., 2011; Marzo et al., 2014) and necrotizing tuberculous pneumonia (Hunter, 2011)], often with a peripheral rim of thrombosed septal capillaries and intra-alveolar neutrophilic debris. By contrast, the lungs of resistant mice typically contained small granulomas, occupying approximately 25-50% of lung tissue, comprising macrophages and abundant perivascular lymphocytes, but little necrosis or neutrophils, resembling the *M.-tuberculosis-*resistant C57BL/6 founder strain (Vesosky et al., 2010; Niazi et al., 2014). Susceptible DO mice showed intermediate patterns with small regions of coagulation and/or caseous necrosis that were surrounded by mixed inflammatory cells (macrophages, lymphocytes, neutrophils), but lacked thrombosis.

These findings link the susceptibility of DO mice to patterns (necrosis, neutrophils) that are similar to those observed in humans with active TB and in other susceptible inbred mice (Barnes et al., 1988; Eruslanov et al., 2005; Berry et al., 2010; Eum et al., 2010; Lyadova et al., 2010; Hunter, 2011; Leong et al., 2011; Harper et al., 2012; Gopal et al., 2013; Major et al., 2013; Hunter et al., 2014). Taken together, these results indicate that lung necrosis and neutrophil influx are common pathways that are likely to reflect multiple different mechanisms or interconnected pathways.

### Lung and peripheral correlates of TB disease in *M.-tuberculosis-*infected DO mice

**Fig. 3.**
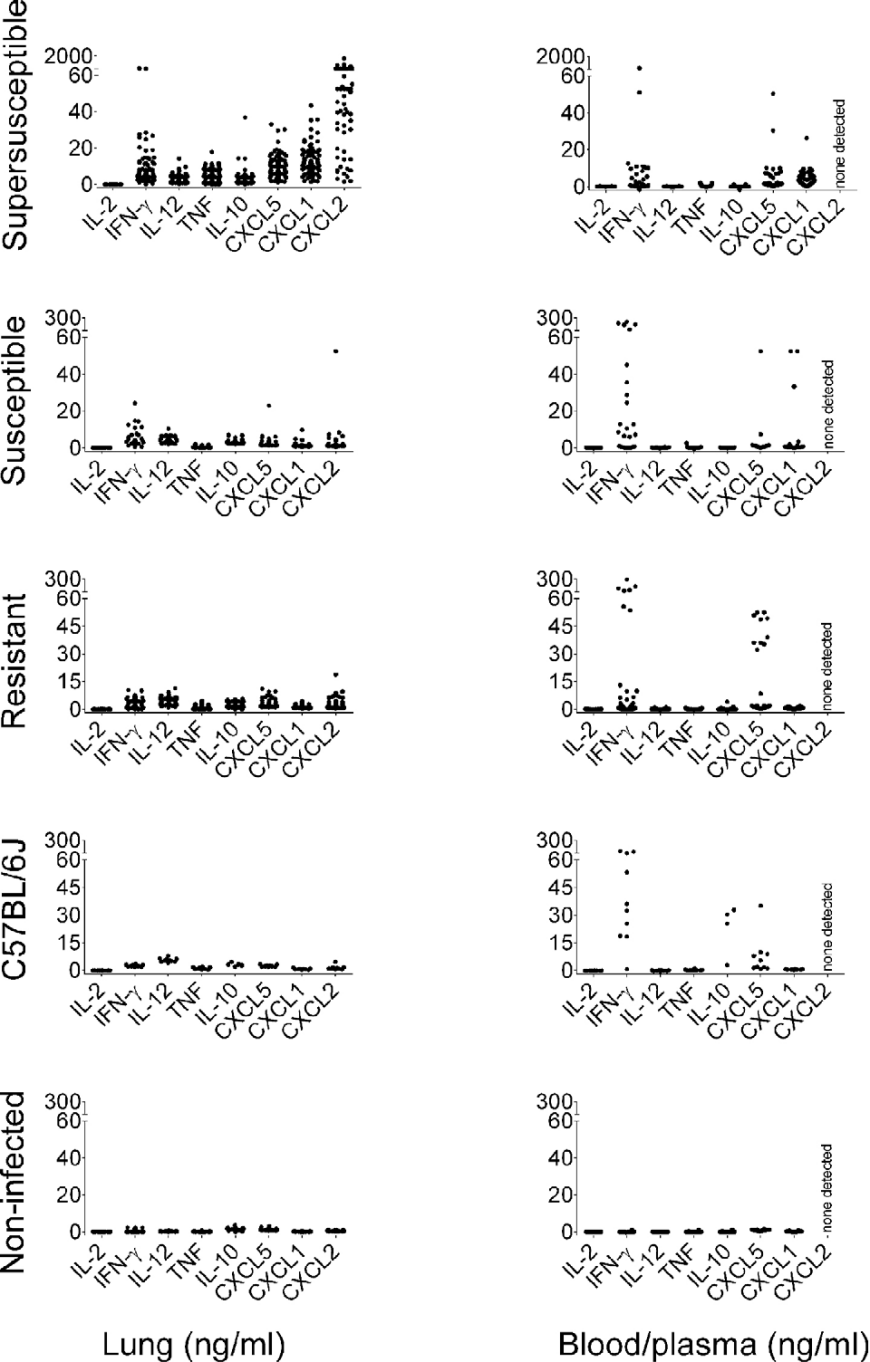
**Molecular profiles of lung, and of blood and plasma in *M.-tuberculosis-*infected mice.** Female 8-week-old non-sibling DO mice (*N*=166) and C57BL/6J (*N*=10) mice were infected with ∼100 *M. tuberculosis* (*M*.*tb*) bacilli by aerosol. Cytokines and chemokines were quantified in homogenized lung. Blood cytokines (IL-2, IFN-γ, IL-12, TNF, IL-10) were quantified after stimulation with antigen. Neutrophil chemokines (CXCL1, CXCL5) were quantified in plasma, but CXCL2 was not detectable. From right to left, molecular features are grouped as follows: T-cell cytokines (IL-2, IFN-γ), macrophage cytokines (IL-12, TNF, IL-10) and neutrophil chemokines (CXCL1, CXCL5, CXCL2). Each dot represents the average of duplicate or triplicate samples from one mouse. The *y*-axes are defined at the bottom of the figure. Data are combined from two independent experiments.

Fig. 3 depicts the cytokine and chemokine data for each class of DO mice (supersusceptible, susceptible, resistant and non-infected) and for the C57BL/6J founder strain for comparison. Using data from infected DO mice, the following chemokines and cytokines were tested for correlations with disease indicators (percentage of peak body weight at euthanasia, bacterial burden, survival): lung, blood and plasma. Lastly, lung and blood and plasma cytokines and chemokines were correlated with each other. Lung cytokine and chemokine correlations with disease indicators are shown in Table 1 [percentage of peak body weight at euthanasia and *M. tuberculosis* colony-forming units (CFU)]. Lung cytokine and chemokine correlations with survival were nearly identical to those for the other indicators (not shown). Statistically significant, very strong or strong disease correlates in DO mouse lungs were dead cells, CXCL1, CXCL2, CXCL5 and TNF, which supports previous studies from inbred mice and humans (Bekker et al., 2000; Gopal et al., 2013; Nouailles et al., 2014). Lung cytokines (IFN-γ, IL-2, IL-12, IL-10) were weak correlates of disease with variable statistical significance.

**Table 1.**
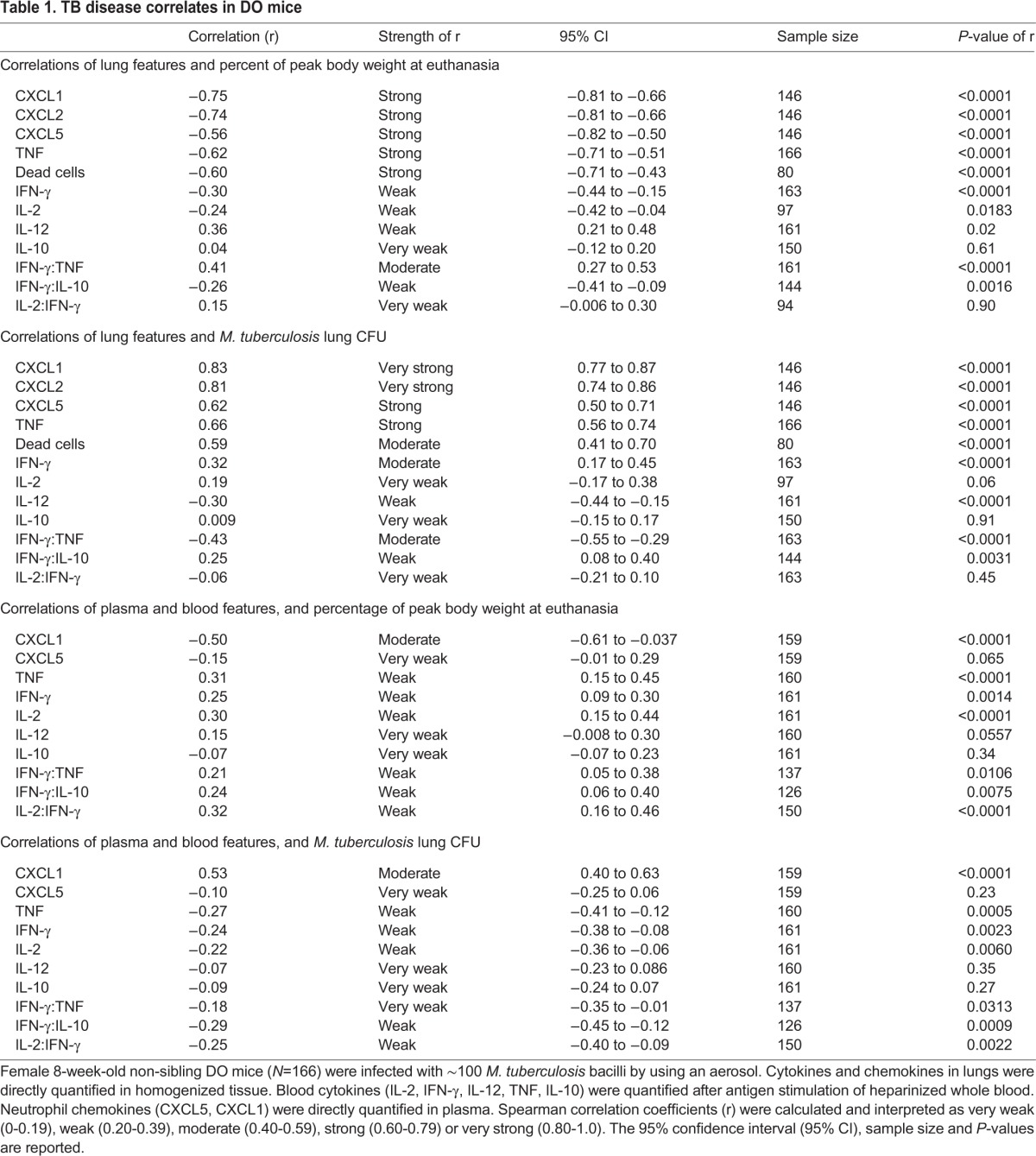
**TB disease correlates in DO mice**

Table 1 also shows correlations of blood and plasma cytokines and chemokines with disease indicators. Plasma CXCL1 was a moderately strong, statistically significant correlate. By contrast, plasma CXCL5 was not a correlate, probably because some resistant mice had high levels (Fig. 3). CXCL2 was not detectable in plasma. Blood cytokines and cytokine ratios (IFN-γ:TNF, IFN-γ:IL-10 and IL-2:IFN-γ) were weak or very weak correlates of disease, although many had highly statistically significant relationships. Importantly, all these results in DO mice mimic studies in humans with TB, such that CXCL1 appears to be a good peripheral biomarker of lung disease (Gopal et al., 2013), and cytokines and the ratios are weak but significant correlates (Jamil et al., 2007; Millington et al., 2007; Sahiratmadja et al., 2007; Suter-Riniker et al., 2011; Hur et al., 2013).

Correlations of lung cytokines and chemokines with those of blood and plasma were also examined. From the molecules that we measured, only plasma CXCL1 correlated with lung CXCL1 levels (supplementary material Fig. S2A) and, as expected from the data presented in Fig. 3, supersusceptible mice had significantly higher levels of CXCL1 in lung and plasma than susceptible, resistant or non-infected DO mice (*P*<0.001, ANOVA with Tukey's post-test). All other cytokines and chemokines, and cytokine ratios had weak or no correlations with the levels in the lungs or blood and plasma (not shown), including CXCL5 (supplementary material Fig. S2B). The paucity of correlations was surprising, so we further probed the relationships of lung cytokine and chemokines with those in the blood and plasma by performing multidimensional analyses [2D-correlation coefficients (Noda, 1993)] and quadratic discriminant analysis (Lachenbruch, 1975; Scholkopft and Mullert, 1999). These analyses also failed to detect significant relationships between lung and blood and plasma profiles, indicating that combined lung and peripheral responses do not mirror each other.

To summarize, the results demonstrate that lung neutrophil chemokines and necrosis are strong and statistically significant TB disease correlates in *M.-tuberculosis-*infected DO mice but that immune cytokines in the lung are not. Of the molecules that we measured, plasma CXCL1 appears to be the best biomarker for neutrophil-associated lung damage, which has been suggested previously in mice and in humans (Gopal et al., 2013).

### Neutrophil chemokines can classify the disease status of *M.-tuberculosis-*infected mice

Statistical analyses and machine learning methods consistently identified similar cytokines and chemokines as being important from the 15 molecular features that we measured. ANOVA followed by Tukey's multiple comparison (MCT) tests identified cytokines and chemokines that could separate pairs of disease classes by using statistical significance (Table 2). These same cytokines and chemokines are inferred from direct observations (Fig. 3) and correlations (Table 1) such that supersusceptible DO mice had high lung TNF, CXCL1, CXCL2 and CXCL5 levels, and a high plasma CXCL1 concentration. Non-infected mice had low to no IFN-γ, IL-12, IL-2 or IL-10. However, ANOVA followed by MCT failed to identify molecules that could distinguish resistant and susceptible mice from each other. A limitation of ANOVA is that some statistical results do not make biological sense. For example, non-infected DO mice could not be identified by using ANOVA and MCT because the confidence interval for *M. tuberculosis* CFUs in resistant mice extended below zero. Thus, although ANOVA followed by MCT corroborated previous results, statistical analyses alone cannot discriminate the four classes of DO mice (supersusceptible, susceptible, resistant and non-infected) because there is no embedded classification method. This led us to use, for the first time in *in vivo* experimental *M. tuberculosis* research, machine learning methods to build testable classification models.

**Table 2.**
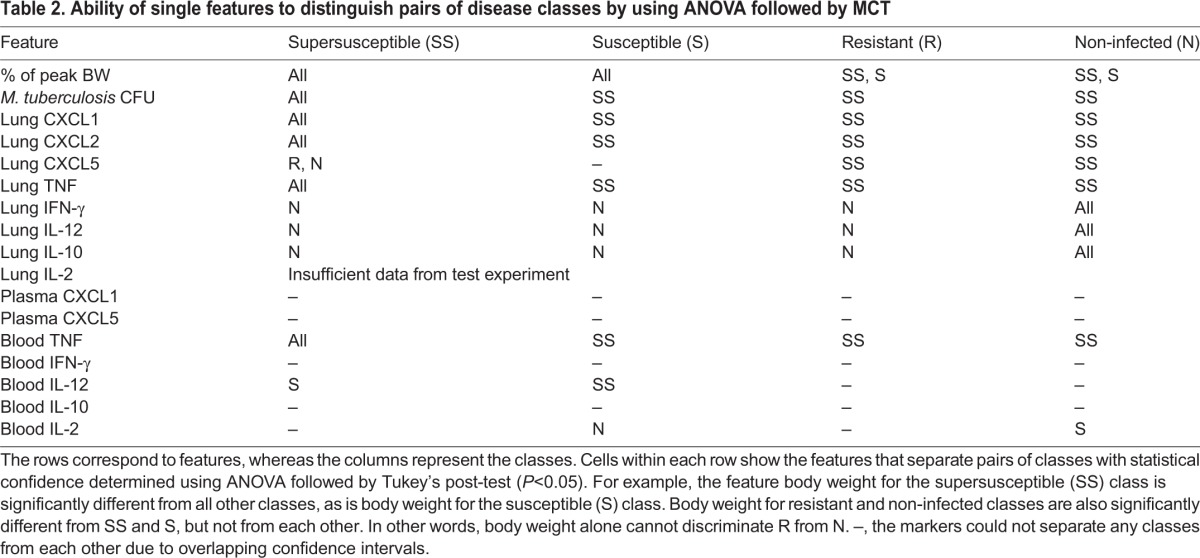
**Ability of single features to distinguish pairs of disease classes by using ANOVA followed by MCT**

**Fig. 4.**
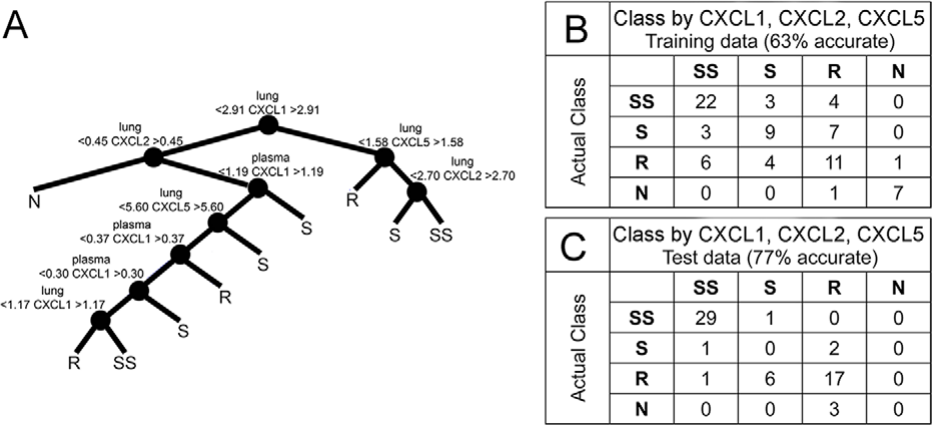
**Classification tree based on neutrophil chemokines CXCL1, CXCL2, CXCL5.** Female 8-week-old non-sibling DO mice and C57BL/6J mice were infected with ∼100 *M. tuberculosis* bacilli by aerosol. From the first experiment, complete data for all 15 molecular parameters used for model generation and training were obtained from 70 *M.-tuberculosis*-infected DO mice and eight non-infected DO mice. The best-performing classification tree used only lung and plasma CXCL1, CXCL2, CXCL5 (A), which was then validated by the leave-one-out method (B). After validation, performance of the classification tree was stringently tested using data from the second independent experiment, which included 52 *M.-tuberculosis*-infected DO mice, three non-infected DO mice and five *M.-tuberculosis*-infected C57BL/6J mice (C), with complete data from all 15 molecular parameters.

By using machine learning methods, we extracted the same molecular features that were strong disease correlates or had some statistical ability to distinguish classes: lung CXCL1, CXCL2, CXCL5, TNF, IFN-γ, IL-12; and two blood features – IL-2 and TNF. Although our data also contained three disease indicators (survival, percentage of peak body weight at euthanasia and *M. tuberculosis* CFU), these were purposefully excluded because the goal was to identify molecules capable of classifying disease status and discrimination of a non-infected state. For building and testing classification models, only data from individual mice with complete data – i.e. all 15 molecular features – were used. Thus, the training and model validation steps used *N*=70 DO mice from the first experiment that had been infected with 127±68 *M. tuberculosis* Erdman bacilli and *N*=8 non-infected DO mice. Multiple machine learning approaches were used, listed in the Materials and Methods. Of these, classification trees were pursued because they produced the most accurate models. Validation for the model used the standard leave-one-out cross-validation for training data (Fig. 4B). However, performance rates in training cannot be generalized. A more stringent approach to evaluate performance (accuracy in classifying disease status of DO and C57BL/6J mice) is to test the model using completely independent molecular data. Therefore, we tested the models using data from a completely independent experiment, again using only mice for which all 15 molecular parameters were available. This included *N*=55 DO mice and *N*=5 C57BL/6J mice from the second experiment that had been infected with 97±61 bacilli and *N*=3 non-infected DO mice.

When tested using independent data, classification trees using the strongest disease correlates [CXCL1, CXCL2, CXCL5 (Fig. 4) and TNF, CXCL1, CXCL2, CXCL5 (not shown)] gave the best results for classifying the disease status of DO mice – 77% and 58%, respectively. Of note, all C57BL/6J mice were correctly classified as resistant by all models. This reflects the widely recognized phenotype of C57BL/6 mice as compared with many other inbred strains of mice (reviewed in Beamer and Turner, 2005). Trees with TNF were problematic because they confused resistant with supersusceptible DO mice, which could reflect the different roles of TNF as a resistance requirement and disease correlate in inbred mice and humans (Law et al., 1996; Bekker et al., 1998, 2000; Juffermans et al., 1998; Ray et al., 2009; Fallahi-Sichani et al., 2010), and in DO mice (Table 1).

All combinations of important molecules were used to generate and test additional classification trees but performance was not improved, ranging between 38 and 61% (Table 3). Models with accuracy of >50% showed TNF to be a dominant feature. Models organized by anatomic site (i.e. lung alone, lung plus blood and plasma, blood and plasma alone) or by function (i.e. immune cytokines IL-2, IL-12, IFN-γ, IL-10) with or without TNF, and with or without CXCL1, CXCL2 and CXCL5 did not improve accuracy above that achieved using CXCL1, CXCL2 and CXCL5 (Fig. 4). Models using cytokine ratios also failed to discriminate supersusceptible, susceptible, resistant and non-infected DO mice (not shown).

**Table 3.**
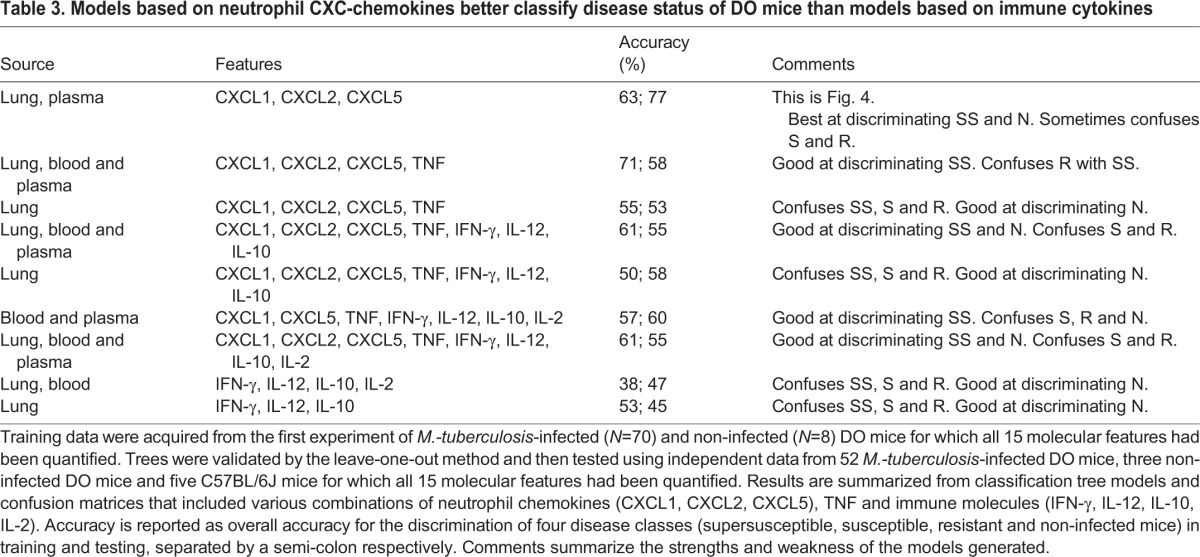
**Models based on neutrophil CXC-chemokines better classify disease status of DO mice than models based on immune cytokines**

To summarize, from the 15 molecule parameters that we measured, the disease status of DO mice and C57BL/6 mice was best classified using three neutrophil chemokines CXCL1, CXCL2 and CXCL5. This classification tree model was particularly good at discriminating supersusceptible DO mice from all other categories, confirming inferences from inspecting raw data (Fig. 3) and statistical analyses (correlations and ANOVA-MCT); this model also has the benefit of being performance-tested with independent data. However, the model was not perfect. It misclassified some resistant and susceptible DO mice, and misclassified non-infected DO mice as resistant in testing. We next investigated whether the format of the data influenced the selection of important molecular features and assessed how DO mice were misclassified.

### Some DO mice that were misclassified on the basis of neutrophil chemokines have unique phenotypes

To determine whether the magnitude of cytokine and chemokine responses influenced feature selection, data were normalized. This had no effect, and the same molecules were identified as being important (not shown).

The numbers of misclassifications were too small to apply data mining in order to extract information, so all results were manually inspected to identify recording errors (none were found) and to seek biologically relevant explanations. Because of the method by which classification tree models are generated, it is not possible to examine ‘misclassifications’ in the training data. Therefore, only misclassifications in the test data were evaluated. Thirteen of the 55 DO test mice were misclassified by using CXCL1, CXCL2, CXCL5 (Fig. 4). Three were non-infected mice that were misclassified as resistant. This error was due to higher lung CXCL2 levels in the test (average 0.8 ng/ml) than the training (average 0.4 ng/ml) data. Thus, these non-infected mice were erroneously classified as resistant because of inter-experiment variability. Additional training data from non-infected mice would reduce this type of misclassification.

Seven resistant DO mice were misclassified. Of these seven, two were misclassified because of high CXCL1 and CXCL5, possibly owing to macrophage production without substantial neutrophil recruitment, which was corroborated by using microscopy analyses. The remaining five resistant mice were misclassified after the first node, despite having typical lung lesions. Three susceptible mice were misclassified after the first node, and these mice had a variety of lung lesions. Only one supersusceptible mouse was misclassified. This mouse was clearly different based on the molecule profile (lung CXCL1 less than 2.91 ng/ml) and microscopy analyses. Instead of neutrophil infiltration and necrosis, morbidity reflected massive lung infiltration by non-necrotic macrophages.

Overall, the examination of misclassified mice was helpful. Misclassified non-infected mice reflected inter-experiment variability, which could be improved with additional training data. Two misclassified resistant mice better fit the susceptible class, showing that the model might help to re-classify ambiguous cases. It was unclear how susceptible mice were misclassified because the numbers were too small to draw meaningful conclusions. The one misclassified supersusceptible mouse had a unique phenotype – marked infiltration by non-necrotic macrophages without neutrophils, which suggests another, but less common, response to *M. tuberculosis* that leads to rapid morbidity.

## DISCUSSION

We investigated survival, morbidity, bacterial load, immune and inflammatory molecules in *M.-tuberculosis-*infected and non-infected DO mice, which is a genetically heterogeneous experimental population. It is another useful mouse model for TB research because the genetic diversity in the DO population mimics the diversity in the human population. The most relevant findings from the studies here are that many of the DO responses to *M. tuberculosis* infection model human responses, including lung and granuloma necrosis and neutrophil influx; production of inflammatory molecules; production of immunological cytokine responses, indicative of antigen-specific TH1 immunity; and production of immune-suppressive cytokines. Furthermore, like humans, neutrophil inflammation and necrosis are strong features of TB disease, whereas immune cytokines are weak or very weak correlates of disease.

We included IFN-γ, IL-12, TNF, IL-2, IL-10 in these DO studies because these cytokines significantly alter survival when absent or blocked in inbred mice and because they have been extensively investigated in humans with TB. Like humans, *M.-tuberculosis-*infected DO mice produce immune cytokines, but these molecules are poor disease correlates and could not be used to discriminate disease status. CXCL1, CXCL2 and CXCL5 were included because CXC-chemokines might contribute to detrimental inflammation and neutrophil influx, or might be biomarkers of lung disease (Gong et al., 1996; Flynn and Chan, 2001; Turner et al., 2002; Beamer et al., 2008a,b; Cooper, 2009; Higgins et al., 2009; Zhang et al., 2011; Gopal et al., 2013; Nouailles et al., 2014). In particular, plasma CXCL1 has been suggested as a good peripheral biomarker of lung disease (Gopal et al., 2013). Likewise, in our studies we observed that lung CXC chemokines strongly correlate with disease and that CXCL1 appears to be the best peripheral biomarker of disease in a genetically diverse population. CXCL5 was not a good peripheral biomarker, and CXCL2 was below the limit of detection in plasma and could not be assessed as a peripheral biomarker.

To further utilize the DO data, we used supervised machine learning methods to generate and test tree classification models with the intent of identifying molecules that could determine TB disease status and identify the non-infected state. The best model successfully discriminated four classes (supersusceptible, susceptible, resistant and non-infected) from each other with 77% accuracy using only CXCL1, CXCL2 and CXCL5. Performance was excellent in distinguishing supersusceptible mice from all other classes, but performed less well in distinguishing susceptible, resistant and non-infected mice. Non-infected mice were misclassified owing to inter-experimental variability, which can be corrected with additional data. The reasons for misclassification of susceptible and resistant mice were not clear, indicating that additional molecular features are needed to improve discrimination.

We also analyzed immune cytokines that are important for resistance (TNF, IFN-γ, IL-12, IL-2) and one cytokine that contributes to susceptibility (IL-10) (Gong et al., 1996; Flynn and Chan, 2001; Turner et al., 2002; Beamer et al., 2008a,b; Cooper, 2009; Higgins et al., 2009; Zhang et al., 2011). All were poor correlates of disease with the exception of TNF, which is likely to reflect its dual role as a marker of resistance and as a disease correlate. All models using immune cytokines from any anatomic location (lung and periphery) failed to classify the disease status of *M.-tuberculosis-*infected DO mice but were very good at discriminating non-infected mice. These findings are important because they reflect meta-analyses from human data and the recent literature reviews by experts, which conclude that a lack of TH1 responses identifies a lack of exposure to *M. tuberculosis*, whereas the presence of TH1 immunity can neither rule in or out TB disease in humans (Modlin and Bloom, 2013; Andersen and Woodworth, 2014; Nunes-Alves et al., 2014; Rangaka et al., 2012). Thus, the DO population could be a good experimental model to help identify better markers of immunological resistance to *M. tuberculosis*.

Performance of the tree classification models could have been improved by computational approaches – such as collapsing the data into two classes (supersusceptible and “other”), replicating data to yield groups of equivalent numbers and combining data into a single training set. However, we did not pursue these approaches because they do not reflect experimental or biological reality. The concept of generating and testing models with independent data might be relevant to human studies where subjects are assigned to clinical categories by TB disease severity (i.e. diagnoses of latent infection, active pulmonary TB, extra pulmonary TB etc.). The responses of each diagnostic category are then compared with each other and analyzed by using statistical methods. However, as we observed here, statistical analyses might not produce testable or accurate models if multiple classes are present and there is abundant or complex data. Therefore, we suggest that machine learning methods are another useful tool to discriminate TB disease status in humans.

DO mice have been used previously in only two studies (Gopal et al., 2013; Harrison et al., 2014), but the breadth of DO responses to *M. tuberculosis* was not the focus. Here, the survival of supersusceptible DO mice mimics that of immune-deficient C57BL/6 mice that cannot produce TH1 responses (reviewed in Flynn and Chan, 2001; Cooper, 2009 and references therein). However, neither DO mice nor the eight founder strains have these immune defects. Furthermore, we demonstrated that DO mice are fully capable of producing TH1 immune cytokines locally and systemically in response to *M. tuberculosis*. Thus, susceptibility in the DO population is not due to inadequate immunity but instead reflects TB disease pathways involving necrosis and neutrophils, and possibly endothelial damage.

The remarkable susceptibility of some DO mice also indicates that genetic material from founder strains enhances susceptibility to *M. tuberculosis*. Based on on-going studies, genetic material associated with increased susceptibility comes from four founder strains – NOD/LtJ, NZO/HlLtJ, PWK/PhJ and WSB/EiJ (Christopher Sassetti, personal communication). However, further research is needed to identify novel susceptibility loci, and the DO population is ideally suited because the genomes are constructed for high-resolution genetic mapping (Zhang et al., 2012; Logan et al., 2013). DO susceptibility should not involve the *Ipr1* polymorphism in C3HeB/FeJ mice (Pan et al., 2005; Tosh et al., 2006; Pichugin et al., 2009) as the polymorphism is absent from DO founders (Mouse Phylogeny Viewer and the Sanger Mouse Genome Database accessed on October 6th 2014 by D.G. in Dr Gary Churchill's laboratory, The Jackson Laboratory, Bar Harbor, ME). Therefore, future genetic studies using DO mice should identify additional genes and loci that contribute to susceptibility.

An important finding from these studies is that neutrophils and neutrophil chemokines are correlates of TB disease in *M.-tuberculosis-*infected DO mice. These same patterns reflect pathologic features of human pulmonary TB. Interest in neutrophil responses and consequences during *M. tuberculosis* infection in human and mouse studies has been increasing slowly over the past few decades and is now accelerating (Barnes et al., 1988; Keller et al., 2006; Eum et al., 2010; Gopal et al., 2013; Major et al., 2013). The majority of studies implicate neutrophils or neutrophil-like cells as causal mediators of lung damage (Keller et al., 2006; Lyadova et al., 2010; Nandi and Behar, 2011; Yang et al., 2012; Gopal et al., 2013; Major et al., 2013; Dorhoi et al., 2014; Nouailles et al., 2014), but the underlying molecular mechanisms and pathways are not yet well defined. The fundamental biological properties of neutrophils as short-lived, innate immune cells that secrete proteases, oxidants, and inflammatory cytokines and chemokines (Elkington et al., 2011; Palanisamy et al., 2011; Amulic et al., 2012; Wilkinson et al., 2012; Gopal et al., 2013; Dorhoi et al., 2014; Nouailles et al., 2014) support this concept that neutrophils are destructive to lung tissue. However, experimental evidence in mouse *M. tuberculosis* studies is not that straightforward. It appears that neutrophils play a variety of roles that differ depending on the host, the dose of *M. tuberculosis* and the duration of *M. tuberculosis* infection. For example, depletion of neutrophils during early infection improves survival of *M.-tuberculosis-*susceptible DBA/2 mice but only with high-dose infection, and the same depletion protocol has no effect on resistant C57BL/6 mice (Keller et al., 2006). This supports the hypothesis that neutrophils are detrimental under certain conditions. By contrast, our unpublished observations indicate that neutrophil depletion during chronic infection accelerates TB disease in CBA/J mice, suggesting that neutrophils are protective under other conditions. Given that some DO mice recruit neutrophils to the lungs and have high levels of CXCL1, CXCL2 and CXCL5, DO mice provide another useful model to understand the signals that attract neutrophils and to define the context(s) in which neutrophils are detrimental or protective. This will be important for a more complete understanding of TB pathogenesis across a genetically diverse population, and also to assess outcomes of interventions aimed at reducing neutrophil-mediated damage.

A limitation of our study could have been the use of light microscopy to identify immune and inflammatory responses in lung sections. This approach maintains the tissue and granuloma architecture, providing a wealth of visual information but at the expense of rigorous identification and quantification of specific immune cells (e.g. T cells, B cells, macrophages, dendritic cells, and all of their subsets etc.) that participate in host responses to *M. tuberculosis*. We did not attempt identification of immature neutrophils (also known as ‘bands’, ‘band cells’ or ‘band neutrophils’) because the morphology might overlap with immature myeloid-derived suppressor cells that have been recently described in lethal murine TB (Tsiganov et al., 2014).

Given the remarkable susceptibility of some DO mice to *M. tuberculosis*, future studies with a lower infectious dose will be important to slow lung damage and to improve detection of individual granulomas. This approach successfully identified multiple susceptibility loci and one gene *Ipr1* (mouse) that alters macrophage necrosis and increases susceptibility, and helped to identify SP110 (the human homolog to mouse *Ipr1*) polymorphisms in TB-infected individuals (Kramnik et al., 1998, 2000; Pan et al., 2005; Tosh et al., 2006; Yan et al., 2006; Kramnik, 2008; Pichugin et al., 2009; Sissons et al., 2009). However, the SP110 polymorphisms do not explain all TB cases (Thye et al., 2006; Png et al., 2012) and additional studies are needed.

A mouse model might not recapitulate all characteristics of human pulmonary TB. However, mice are important animals to investigate experimental questions that cannot be addressed in humans. Here, DO mice are advantageous because the population provides immunologically intact, genetically diverse individuals, and the phenotypic disease spectrum overlaps with humans. Using machine learning to build models that can classify disease status is new and useful, particularly where statistical analyses are not successful. In this context, machine learning will be especially useful to move beyond classification of TB disease status (which we describe here for the first time using only molecular correlates of disease) to studies that are capable of predicting TB disease outcome before it happens. That is a powerful approach and is achievable by sampling individual *M.-tuberculosis-*infected DO mice over time using peripheral host and mycobacterial biomarkers, followed by data mining, machine learning and independent testing.

## MATERIALS AND METHODS

### Ethics statement

The Cummings School of Veterinary Medicine (CSVM) at Tufts University has a letter of Assurance on file with the Office of Laboratory Animal Welfare. CSVM follows the Public Health Service Policy on Humane Care and Use of Laboratory Animals; the Guide for the Care and Use of Laboratory Animals; and the U.S. Government Principles for the Utilization and Care of Vertebrate Animals Used in Testing, Research and Training. CSVM is also accredited by AAALACi, complies with the USDA Animal Welfare Regulations and is a USDA APHIS registered Research Facility (14-R-0065). Investigators follow guidelines pertinent for the species. Here, experimental procedures were approved by Tufts University IACUC protocols (G2012-53; G2012-151; G2015-33). Biosafety Level 3 (BSL3) work was approved by Tufts University Institutional Biosafety Committee registration (GRIA04; GRIA10).

### Infection with *Mycobacterium tuberculosis* and quantification of lung bacilli

Female J:DO (009376) and C57BL/6J (000664) mice (The Jackson Laboratory, Bar Harbor, ME, USA) were maintained under BSL3 conditions with sterile food, bedding and water in the New England Regional Biosafety Laboratory (North Grafton, MA, USA). At 8 weeks of age, mice were infected by aerosol exposure using a CH Technologies, Inc (Westwood, New Jersey, USA) machine. Exposure delivered 127±68 *M. tuberculosis* Erdman bacilli to the lungs (*N*=97 DO mice) that resulted in complete data from 70 individual DO mice to train machine learning models. A separate experiment delivered 97±61 bacilli to the lungs (*N*=69 DO and *N*=10 C57BL/6J mice) that resulted in complete data from *N*=52 DO mice and *N*=5 C57BL/6J mice to test machine learning models with the independent molecular data. Lung bacillary burden was determined following homogenization in sterile PBS using gMACS M-tubes (Miltenyi Biotech, Cambridge, MA, USA), plating serial dilutions on OADC-supplemented 7H11 agar and counting CFUs after 3 weeks at 37°C.

### Identification of disease classes

Mice were euthanized when morbidity met IACUC-approved early removal criteria, or at day 35 of infection, whichever came first. Morbidity criteria included: weakness, respiratory difficulty, ruffled fur and a body condition score of less than 2 (Ullman-Cullere and Foltz, 1999). Body weight was monitored throughout, but weight loss alone was not sufficient for early euthanasia nor was body weight used as a threshold. Mice that met early removal criteria before day 35 were classified as supersusceptible. Susceptible mice had no signs of morbidity before day 35, but retrospective analysis identified some weight loss. Resistant mice survived 35 days of *M. tuberculosis* infection without any signs of morbidity, and retrospective analysis identified stable weight or weigh gain. Age- and sex-matched non-infected control mice, housed identically, were euthanized on day 35. Supersusceptible and resistant classes were fundamentally based on survival, whereas susceptible mice were those that survived to day 35 without external evidence of morbidity but had lost weight upon retrospective analysis. These classes and non-infected DO mice (*N*=8 for model training and *N*=3 for model testing) were used as the ground truth for establishing and training algorithms.

### Cytokine measurements

ELISAs for TNF, IFN-γ, IL-12, IL-2 and IL-10 were performed using antibody pairs and standards or OptEIA kits (BD Biosciences, San Jose, CA, USA) on serially diluted homogenized lung or from antigen-stimulated whole blood as described, with the exception that blood was diluted 1:5 (Beamer et al., 2008a,b). Samples at or below the IFN-γ level of detection were repeated using the eBioscience Ready-Set-Go ELISPOT kit (San Diego, CA, USA) (Beamer et al., 2011). CXCL1, CXCL2 and CXCL5 were quantified using R&D Systems ELISA kits (Minneapolis, MN, USA) on diluted homogenized lung and plasma.

### Enumeration of dead cells

Dead cells were enumerated following collagenase and DNase digestion of the lungs, and trypan blue staining on single cell suspensions (Beamer et al., 2012).

### Light microscopy

Lung lobes were inflated and fixed with 10% neutral buffered formalin, processed, embedded in paraffin, cut at 5 µm and stained with hematoxylin and eosin at the CSVM Histology Laboratory. Two serial sections, 100 µm apart, were examined by a board-certified veterinary pathologist (G.B.) without knowledge of the groups with regards to the relative extent of necrosis, neutrophils, macrophages and lymphocyte influx, and estimates of the proportion of effaced lung tissue.

### Statistical analyses

GraphPad Prism 6.0 was used for correlations. Data had non-normal distributions, so Spearman correlation coefficients were calculated and identified as very weak (0-0.19), weak (0.20-0.39), moderate (0.40-0.59), strong (0.60-0.79), or very strong (0.80-1.0) and considered statistically significant if *P*<0.05. MATLAB^®^ was used for analysis of variance (ANOVA) followed by Tukey's MCT to identify which features had significantly different means between any two classes, defined as *P*<0.05.

### Machine learning

Machine learning can help identify complex relationships by exploring data in multidimensional space (Tibshirani, 1996; Kornaropoulos et al., 2014). We applied standard machine learning methods for model training and testing, using mice with all parameters measured (*N*=78 mice for training and *N*=60 mice for testing). Five supervised [classification tree, relief attribute evaluation, consistency subset, spectral feature selection and a custom principal components algorithm (Kira and Rendell, 1992; Liu and Setiono, 1996; Coppersmith et al., 1999; Rokach and Maimon, 2005; Zheng and Liu, 2007)]; and two unsupervised [brute force and standard principal component analysis (Boutsidis et al., 2008)] methods were used. Of all methods, classification trees and relief attribute evaluation were the most informative because these methods ranked molecular features by importance. Classification trees were pursued for validation and testing with independent cytokine and chemokine molecular data because these trees provided the most accurate models.

## Supplementary Material

Supplementary Material
