# Supplementary material for: Lung necrosis and neutrophils reflect common pathways of susceptibility to Mycobacterium tuberculosis in genetically diverse, immune-competent mice
Source: Dis Model Mech. 2015 Sep 1;8(9):1141–53. doi: 10.1242/dmm.020867 (PMC4582107; doi:10.1242/dmm.020867)
Supplement: Supplementary Material [file supp_8_9_1141__index.html]

Supplementary Material 

# Lung necrosis and neutrophils reflect common pathways of susceptibility to *Mycobacterium tuberculosis* in genetically diverse, immune-competent mice

## DMM020867 Supplementary Material

- Supplementary Material
