# Supplementary material for: Lung necrosis and neutrophils reflect common pathways of susceptibility to Mycobacterium tuberculosis in genetically diverse, immune-competent mice
Source: Dis Model Mech. 2015 Sep 1;8(9):1141–53. doi: 10.1242/dmm.020867 (PMC4582107; doi:10.1242/dmm.020867)
Supplement: Supplementary Material [file supp_020867_DMM020867supp.pdf]

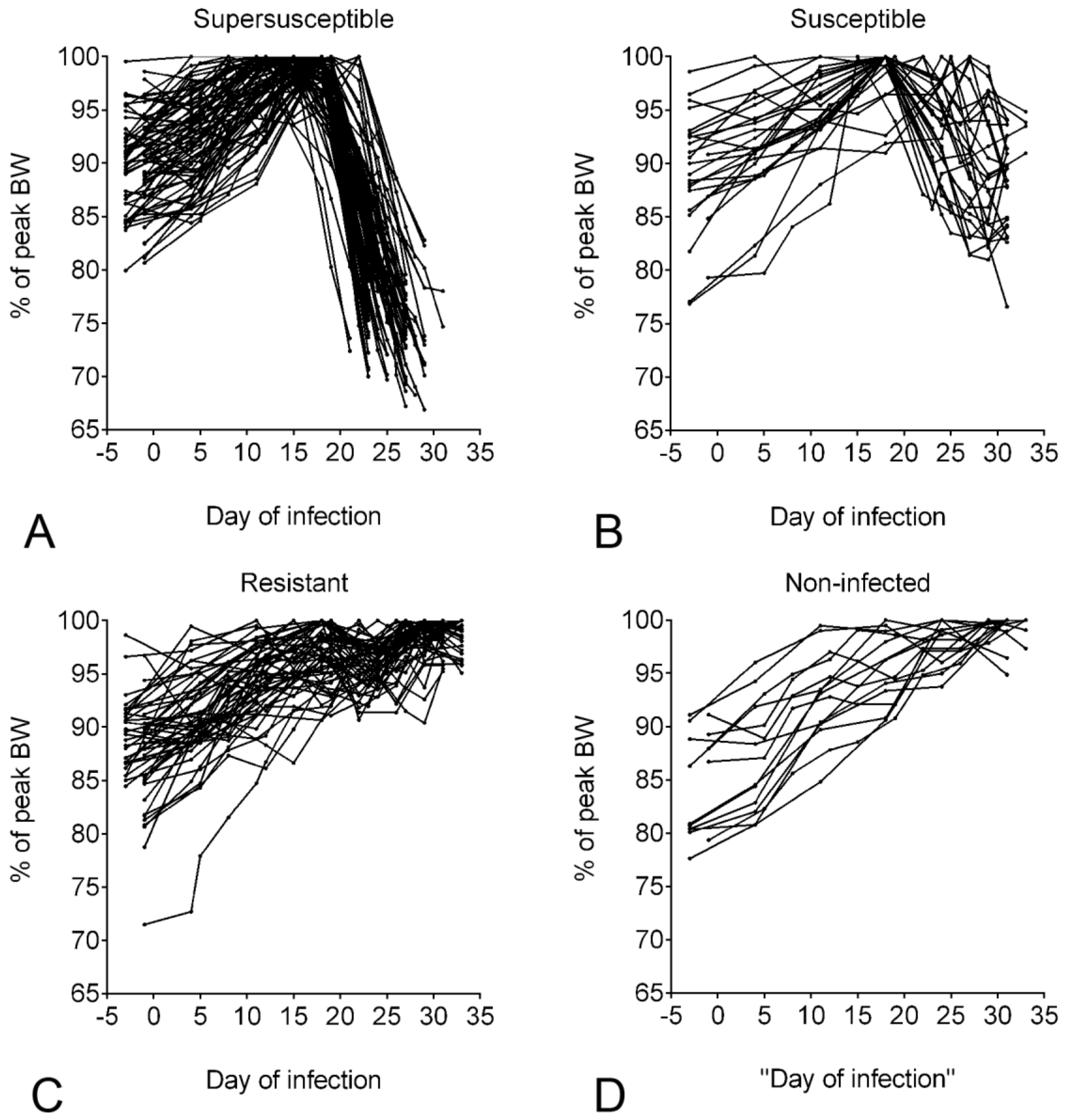

Fig. S1. Longitudinal measurements of body weight in mice. Female, non-sibling, DO mice were weighed 1 to 3 days prior to infection with ~100 *M. tuberculosis* bacilli by aerosol. After infection mice were weighed two to three times per week, with the frequency accelerated to daily if weight was lost for three consecutive measurements. Euthanasia followed IACUC early removal criteria based on clinical indicators and not on weight loss alone. The early removal criteria were included: weakness, respiratory difficulty, ruffled fur, and body condition score of less than 2 (Ullman-Cullere and Foltz, 1999). Mice that met early removal criteria before day 35 were classified as Supersusceptible (A). Susceptible mice did not meet early removal criteria before day 35, but retrospective analysis identified some weight loss (B). Resistant mice survived 35 days of *M. tuberculosis* infection without any signs of morbidity, and retrospective analysis identified stable weight or weigh gain (C). Age- and sex-matched Noninfected control mice, housed identically, were euthanized on day 35 (D). These classes were used as the ground truth for building and testing models.

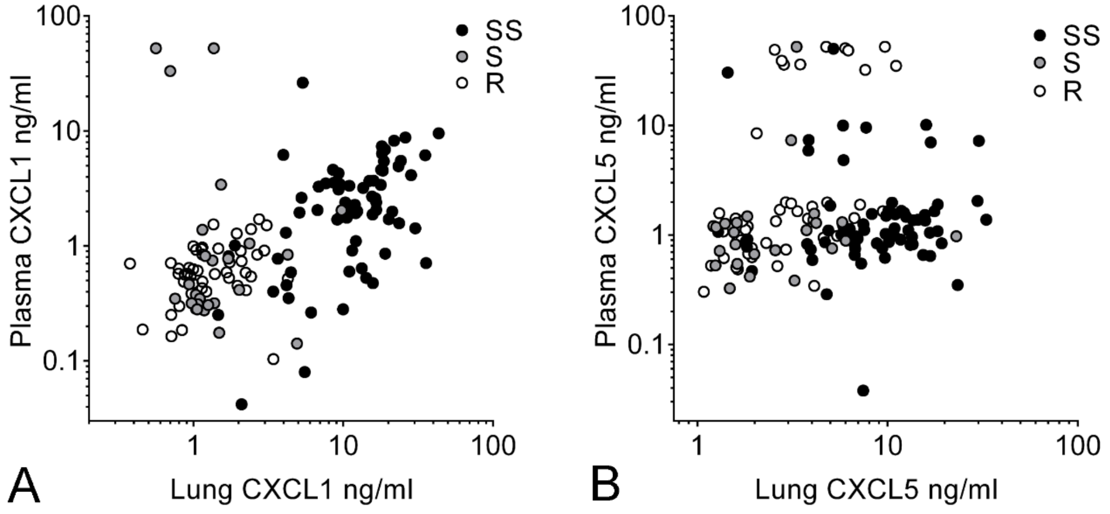

Fig S2. Correlations between lung and plasma CXC neutrophil chemokines. 8-week-old, female, non-sibling, DO mice (*N*=166) were infected with ~100 *M. tuberculosis* bacilli by aerosol. CXCL1 and CXCL5 lung and plasma levels were quantified by ELISA from samples collected at the time of euthanasia for Supersusceptible (SS), Susceptible (S), and Resistant (R) DO mice. Correlation analyses identified a strong, highly statistically significant relationship between CXCL1 in lungs and plasma (Spearman r 0.6, *P*<0.0001) (A) and very weak, marginally statistically significant relationship between CXCL5 in lungs and plasma (Spearman r 0.16, *P*<0.0458).
